# Controlled synthesis of dicalcium phosphate dihydrate (DCPD) from metastable solutions: insights into pathogenic calcification

**DOI:** 10.1007/s10856-021-06617-4

**Published:** 2021-11-24

**Authors:** A. D. Rafeek, G. Choi, L. A. Evans

**Affiliations:** School of Mathematical and Physical Sciences, Faculty of Science, University of Technology Sydney, Ultimo, NSW Australia

**Keywords:** Dicalcium phosphate dihydrate, Calcium pyrophosphate, Seeded growth, pH-stat, Metastable solution

## Abstract

Calcium phosphate (CaP) compounds may occur in the body as abnormal pathogenic phases in addition to their normal occurrence as bones and teeth. Dicalcium phosphate dihydrate (DCPD; CaPO_4_·2H_2_O), along with other significant CaP phases, have been observed in pathogenic calcifications such as dental calculi, kidney stones and urinary stones. While other studies have shown that polar amino acids can inhibit the growth of CaPs, these studies have mainly focused on hydroxyapatite (HAp; Ca_10_(PO_4_)_6_(OH)_2_) formation from highly supersaturated solutions, while their effects on DCPD nucleation and growth from metastable solutions have been less thoroughly explored. By further elucidating the mechanisms of DCPD formation and the influence of amino acids on those mechanisms, insights may be gained into ways that amino acids could be used in treatment and prevention of unwanted calcifications. The current study involved seeded growth of DCPD from metastable solutions at constant pH in the presence of neutral, acidic and phosphorylated amino acid side chains. As a comparison, solutions were also seeded with calcium pyrophosphate (CPP; Ca_2_P_2_O_7_), a known calcium phosphate inhibitor. The results show that polar amino acids inhibit DCPD growth; this likely occurs due to electrostatic interactions between amino acid side groups and charged DCPD surfaces. Phosphoserine had the greatest inhibitory ability of the amino acids tested, with an effect equal to that of CPP. Clustering of DCPD crystals giving rise to a “chrysanthemum-like” morphology was noted with glutamic acid. This study concludes that molecules containing an increased number of polar side groups will enhance the inhibition of DCPD seeded growth from metastable solutions.

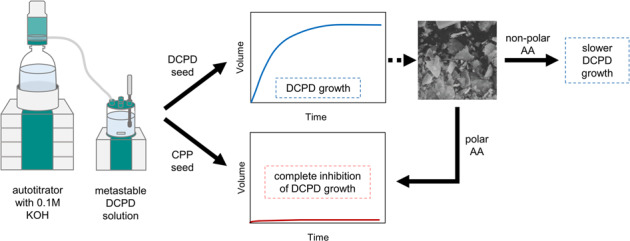

## Introduction

For a solution to be supersaturated it must contain more than the maximum level of solute at a given temperature [1]. Spontaneous crystallisation from these solutions will occur if the free energy of the solution is higher than that of the precipitating phase. This causes monomer units (ions, atoms and molecules) to cluster together to form nuclei. These nuclei have high interfacial energy (∆*G*_*i*_) barriers that can only be overcome when they grow greater than a critical size (*n*_*crit*_). When this is achieved ∆*G*_*i*_ becomes insignificant in comparison to the energy reduction associated with the formation of bonds in the new bulk phase (∆*G*_***B***_). The magnitude of *n*_*crit*_ is proportional *to* $\frac{{\Delta G_i}}{{\Delta G_B}}$, while ∆*G*_***B***_ is proportional to relative supersaturation (*S*) [2]. This relationship is seen in the Eq. (1):

1 $$\Delta G_B = {\it{kTlog}}_eS$$

where *k* is the Boltzmann constant, *T* is absolute temperature. The equation for *S* is given in Eq. (2):

2 $${{{{{\mathrm{S}}}}}} = {\it{relative}}\,{\it{supersaturation}} = \frac{{{\it{ionic}}\,{\it{concentration}}\,{\it{of}}\,{\it{solution}}\,\left( {\it{c}} \right)}}{{{\it{ionic}}\,{\it{concentration}}\,{\it{at}}\,{\it{equilibrium}}\,\left( {{\it{c}}_{\it{0}}} \right)}}$$

Thus, it can be concluded that:

3 $$n_{{{{{{\mathrm{crit}}}}}}} \propto \frac{1}{S}$$

Equation (3) demonstrates that as supersaturation increases, critical nuclei size decreases. It might therefore seem advantageous to precipitate calcium phosphates (CaPs) from highly supersaturated solutions, however some studies have shown that this can lead to uncontrolled crystallisation [3]. Low *n*_*crit*_ values can lead to small particle sizes making filterability difficult. High levels of crystal aggregation are problematic, resulting in varied morphologies and large particle size distributions [4]. Rodríguez-Clemente et al. hypothesised that aggregation occurs to release excess surface free energy (*G*_*s*_) in solutions containing residual supersaturation (supersaturation in excess of the critical level needed for nucleation) [5]. When aggregation occurs, crystal surface areas decrease, as does *G*_*s*_. High supersaturations, however, differ greatly from the low supersaturations present in vivo, thus offering little insight into biological processes such as those found in pathogenic calcifications like dental calculi and kidney stones.

### Dental calculi

Dental calculus is the mineralised plaque that forms on and above the gum line. If left untreated, calculi can lead to serious oral health problems such as periodontal disease. A biofilm composed of salivary proteins forms on tooth enamel immediately after cleaning. This is called a pellicle and prevents further CaP crystal growth. When exposed to saliva, bacteria colonise on the pellicle, which is then referred to as plaque and if not removed begins to mineralise [6]. The exact mechanisms of this mineralisation process are not known, however, it is thought to be facilitated by pH, proteins, and calcium and phosphate concentrations in saliva [7].

Calculus is composed of 70–80% inorganic material, in which several different CaP compounds have been identified [8]. Phases include carbonate-substituted hydroxyapatite (cHAp; Ca_10_(PO_4_, CO_3_)_6_(OH)_2_), amorphous calcium phosphate (ACP; Ca_*x*_H_*y*_(PO_4_)_*z*·_*n*H_2_O (*n* = 3–4.5)), dicalcium phosphate dihydrate (DCPD; CaPO_4_·2H_2_O), octacalcium phosphate (OCP; Ca_8_H_2_(PO_4_)_6_·5H_2_O), tricalcium phosphate (TCP; Ca_3_(PO_4_)_2_)) and whitlockite (β-TCP; Ca_3_(PO_4_)_2_). DCPD is the first phase to nucleate in the formation of dental calculus and usually transforms to a more stable phase after ~3 days [8].

### Kidney and urinary stones

Pathogenic stones are inorganic crystalline aggregates containing ~5% organic material. They consist predominantly of calcium oxalate monohydrate (COM; C_2_H_2_CaO_5_) with small amounts of CaP [9]. Roughly 15% of stone formers produce pure CaP stones; of these, a quarter are composed of DCPD. These stones are exceptionally hard and are difficult to remove using standard treatments such as shock wave lithotripsy. Studies have indicated that DCPD stone recurrence is high and surgical interventions may be necessary for their removal [10].

While studies have been performed showing the effects of amino acids on HAp crystal growth [11], very few studies exist on the effects of amino acids on DCPD. Thus the aim of the current work was to investigate the effects of organic growth modifiers on the synthesis of CaPs from solutions metastable with respect to DCPD at constant pH. An autotitrator operating in the pH-stat mode was used to monitor the volume of base needed to maintain constant pH as reaction proceeds. Characterisation of both seed and product materials was achieved using the combined techniques of Fourier-transform infra-red (FTIR) and Raman spectroscopy, powder X-ray diffraction (XRD) analysis and scanning electron microscopy (SEM).

## Materials and methods

### Preparation of seed materials

Preparation of highly crystalline DCPD has been described elsewhere [12]. Briefly, this involves the addition of potassium hydrogen phosphate (K_2_HPO_4_) solution to a solution of calcium chloride dihydrate (CaCl_2_·2H_2_O) at elevated pH. The solution is stirred and allowed to age for at least one fhour before filtering through a sintered glass crucible. CPP was prepared from DCPD via heat treatment at 500 °C (Tetlow furnace) [13].

### Solution preparation and pH-stat experiments

All starting materials were obtained from Sigma-Aldrich (Castle Hill, Australia). All solutions were purged with nitrogen (Enware Australia Pty Ltd; Caringbah, Australia) to expel carbon dioxide. Solutions were stored in Pyrex Schott bottles, then sealed with Teflon tape. If not used within three days, fresh solutions were prepared. In order to prevent nucleation by foreign particles, all glassware was rinsed with deionised water and then soaked in a 5% solution of Lab Power (WestLab; Mitchell Park, Australia) cleaning agent for a minimum of 12 h. After this time, glassware was rinsed with deionised water and further cleaned using concentrated nitric acid (HNO_3_). A thorough final rinse with ultrapure water was then performed before drying in a 60 °C oven. A Metrohm 907 Titrando autotitrator was used to accurately control solution pH. This device consists of Ag/AgCl pH electrode with an inbuilt temperature sensor (Unitrode with Pt 1000) connected to an autoburette (800 Dosing unit), controlled using Tiamo^TM^ 2.5 software. Solution pH, temperature and volume of titrant added were recorded every 20 s.

A modified method based on that of Lagno et al. was followed for the preparation of metastable solutions [14]. To prepare solutions which were undersaturated with respect to DCPD, 40 mL of 40 mM KH_2_PO_4_ solution was placed into a 150 mL glass reaction vessel, the solution pH lowered to 3.0 using 0.1 M HCl, and then 40 mL of 40 mM CaCl_2_·2H_2_O solution was added dropwise from a glass burette. At this point, any organic modifiers were added to give a concentration of 40 mM. To bring the solution into the metastable zone, the pH-stat auto titrator (in pH-set mode) delivered 0.75 M KOH solution at a rate of 20 µL/min raising the pH to 5.0. After 30 min, 50 mg of DCPD seed material was added (50 mg of CPP instead of DCPD in experiment E). Once precipitation was initiated by the seed, the pH-stat autotitrator (in pH-stat mode) automatically controlled the pH at pH = 5.000 ± 0.015.

A summary of the experiments performed in the DCPD metastable and labile zones are shown in Table 1. Experiment F was prepared under spontaneous growth conditions with pH initially raised to 10.0 and then allowed to drift.

**Table 1:** Summary of experiments carried out in DCPD metastable and labile zones

Experimental ID	Growth zone	pH	Seed material	Organic modifier
A	metastable	5.0	DCPD	-
B	metastable	5.0	DCPD	alanine
C	metastable	5.0	DCPD	glutamic acid
D	metastable	5.0	DCPD	phosphoserine
E	metastable	5.0	CPP	-
F	labile	drift	-	-

- No seed material or organic modifier used

All reactions were conducted for ~3 h. After this time, solutions were vacuum filtered, washed with 95% ethanol and then dried at 60 °C. Once dried, samples were weighed using an analytical balance, placed in scintillation vials and stored in a desiccator.

### Materials characterisation

FTIR analysis was performed using a Thermo-Scientific Nicolet 6700 instrument using ATR (attenuated total reflectance) mode with a Smart iTX ATR diamond plate. Spectra were collected over 64 scans at a resolution of 4 cm^−1^ and range of 4000–500 cm^−1^. Laser Raman spectroscopy was performed using a Renishaw inVia spectrometer coupled to a Leica DMLB microscope. A spectral range of 400–1800 cm^−1^ was covered using a 633 nm laser. Powder XRD analysis was performed using a Bruker D8 Discover diffractometer with Cu-Kα radiation generated at 40 kV and 40 mA. Diffraction patterns were recorded from 5–50° 2*θ*, using a step size of 0.2° every 0.3 s. All samples were mounted on circular glass coverslips and attached to XRD sample holders. Glass coverslips and powders were attached and prepared such that they were the same height as the XRD sample holders. SEM analysis was performed using a Zeiss EVO LS15 scanning electron microscope operating with an accelerating voltage of 15.00 kV. Samples were mounted on aluminium stubs using carbon adhesive.

## Results

### Characterisation of seed materials

**Fig. 1:**
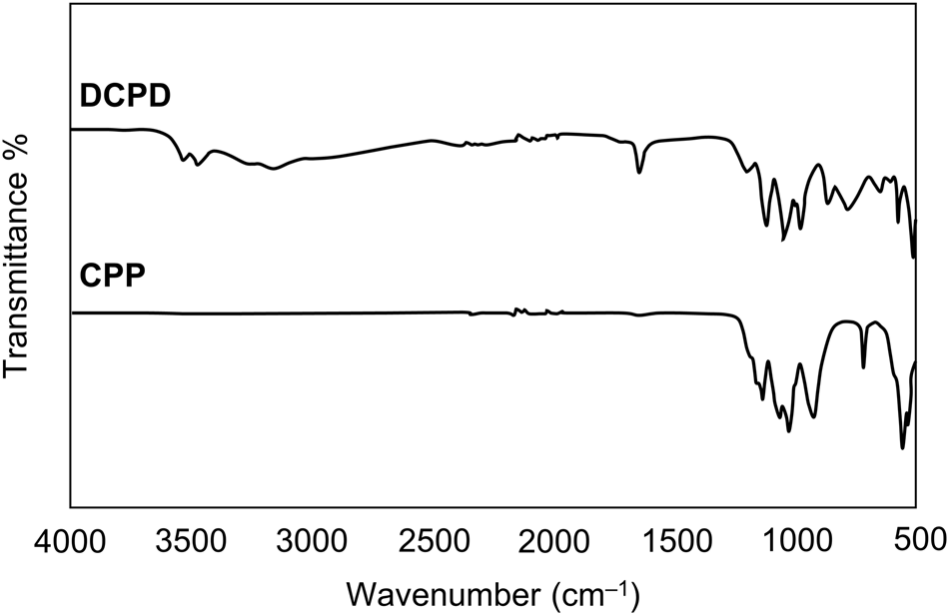
FTIR spectra of DCPD and CPP seed material

**Fig. 2:**
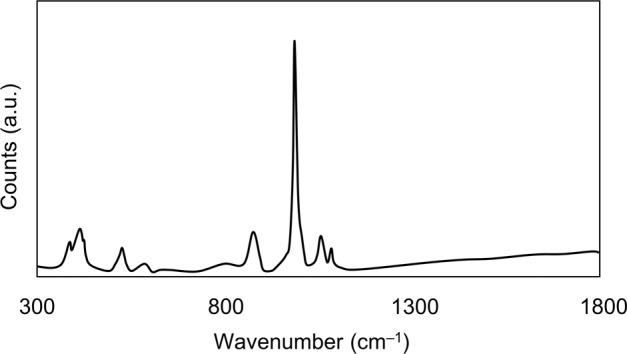
Raman spectrum of DCPD seed material

FTIR and Raman spectra of DCPD and CPP seed materials are shown in Figs. 1 and 2, respectively. The Raman spectrum for CPP could not be obtained due to fluorescence of unknown origin.

**Fig. 3:**
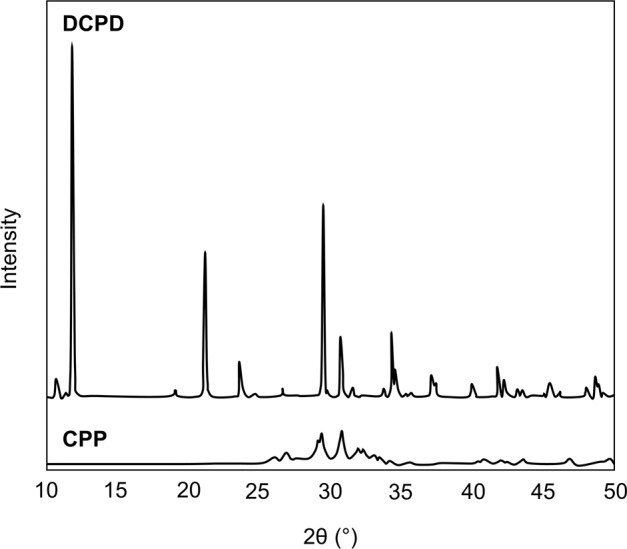
XRD diffraction patterns of DCPD and CPP seed material

Key features of the FTIR spectrum of DCPD seed material are the occurrence of two characteristic OH^−^ stretching doublets: one with components at about 3529 and 3470 cm^−1^ and the other with components at about 3250 and 3155 cm^−1^. The two doublets have different shapes—the higher-wavenumber doublet consists of sharp bands, but the lower-wavenumber doublet is much broader. These bands originate from water of crystallisation in the lattice structure. Medium intensity peaks at 1646 and 782 cm^−1^ can be attributed to H_2_O bending and librational modes respectively. Characteristic phosphate bands also appeared as a triplet at 1124, 1051 and 982 cm^−1^, while P-OH in-plane bending and stretching bands were present at 1204 and 871 cm^−1^ [15, 16]. Confirmation of the phase and purity of the seed material is also provided by the Raman spectrum of DCPD which shows the characteristic shift of the symmetric phosphate stretch from 960 cm^−1^ for HAp to 985 cm^−1^ for DCPD [17]. The powder X-ray diffraction pattern of DCPD seed (Fig. 3) demonstrates a high level of crystallinity as exhibited by the sharpness of the peaks. Major peaks were identified at 11.8, 21.1 and 29.5 2*θ*° corresponding to crystallographic planes (020), (12$\bar 1$) and (11$\bar 2$; 14$\bar 1$). The diffraction pattern did not show the presence of any other CaP phases.

DCPD converts to CPP upon heating, resulting in the loss of two OH doublets in the infra-red spectrum (Fig. 1). A sharp band is apparent at 719 cm^−1^, which is due to the vibration of the P_2_O_7_^4−^ group, revealing that transformation is complete. Other bands of interest include absorptions at 1138, 1071, 1031 and 927 cm^−1^ caused by PO_4_^3−^ vibrations [18]. Anhydrous CPP can exist in either an α, β or γ form (which differ in the way the atoms are arranged in the crystal lattice), depending on the temperature at which heat treatment is performed. The best match of the experimental data with that of the relevant powder diffraction files indicated that the seed material is γ-CPP (JCPDS-17-499). The broadness of the XRD peaks for CPP compared to DCPD is indicative of decreased crystallinity of the CPP seed compared to DCPD.

**Fig. 4:**
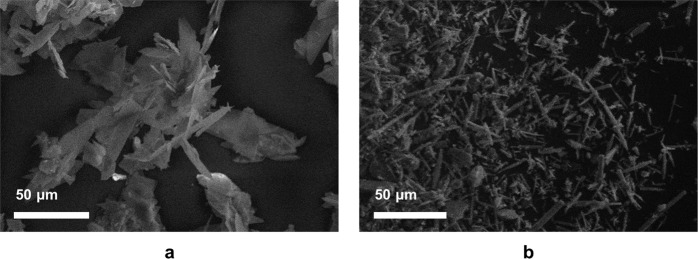
SEM images of DCPD and CPP seed material

SEM images of the two seed materials (Fig. 4) show that DCPD has a plate-like morphology of ~50 μm in length; this morphology is commonly observed for DCPD materials which have been prepared using wet chemical synthesis methods [16, 19]. CPP displayed an acicular rod-like morphologyof ~20 to 30 μm in length. While studies have shown that hydrated CPP phases display acicular, rhombohedral and oval platelets, few studies exist regarding the morphologies of anhydrous CPPs [7, 20, 21]. Crystal morphology, and in particular greater crystal surface area to volume ratio, may enhance the ability to induce growth in metastable solutions as more potential sites for nucleation will be present.

### pH-stat studies

**Fig. 5:**
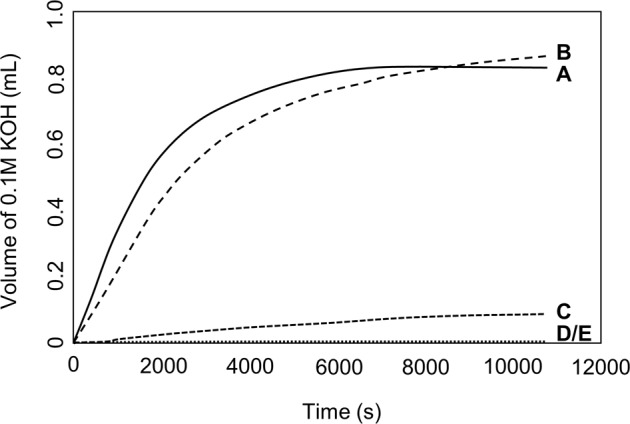
Volume 0.1 M KOH solution vs time curves for experiments performed in the DCPD metastable zone. **A** growth of DCPD on DCPD seed material; **B** growth of DCPD on DCPD seed material in the presence of alanine; **C** growth of DCPD on DCPD seed material in the presence of glutamic acid; **D** growth of DCPD on DCPD seed material in the presence of phosphoserine; **E** growth of DCPD on CPP seed material

Volume/time curves for products grown in the DCPD metastable zone are shown in Fig. 5. Sample F was grown from spontaneous uncontrolled growth conditions and is therefore not referred to in this figure. The interpretation of DCPD curves may assist in understanding the growth of pathogenic CaPs.

Curves A (DCPD seed only) and B (DCPD + alanine) show a steep rise followed by a period where base consumption is slower and more constant; this rise is delayed somewhat in the presence of alanine. Studies by Marshall et al. suggest that the steep rise is due to DCPD nucleation, where the following equilibria in solution take place [22]:

$${{{{{\mathrm{H}}}}}}_2{{{{{\mathrm{PO}}}}}}_4^ - \rightleftharpoons {{{{{\mathrm{H}}}}}}^ + + {{{{{\mathrm{HPO}}}}}}_4^{2 - }$$

$${{{{{\mathrm{Ca}}}}}}^{{{{{{\mathrm{2}}}}}} + } + {{{{{\mathrm{H}}}}}}_2{{{{{\mathrm{PO}}}}}}_4^ - \rightleftharpoons {{{{{\mathrm{CaH}}}}}}_2{{{{{\mathrm{PO}}}}}}_4^{2 + }$$

$${{{{{\mathrm{Ca}}}}}}^{{{{{{\mathrm{2}}}}}} + } + {{{{{\mathrm{HPO}}}}}}_4^{2 - } \rightleftharpoons {{{{{\mathrm{CaHPO}}}}}}_{{{{{\mathrm{4}}}}}}$$

In the latter part of the titration curve crystal growth dominates, and the specific surface area of crystals are thought to increase substantially [22].

Titration curve C (DCPD + glutamic acid) shows inhibition of crystal growth while curves D (DCPD + phosphoserine) and E (CPP seed—no organic modifier) showed complete inhibition of crystal formation. Thus the inhibitory ability of the amino acids can be ranked as follows:

$${{{{{\mathrm{phosphoserine}}}}}}\left( { \approx {{{{{\mathrm{CPP}}}}}}} \right) \, > \, {{{{{\mathrm{glutamic}}}}}}\,{{{{{\mathrm{acid}}}}}} \, > \, {{{{{\mathrm{alanine}}}}}}$$

### FTIR and laser Raman spectroscopy

**Fig. 6:**
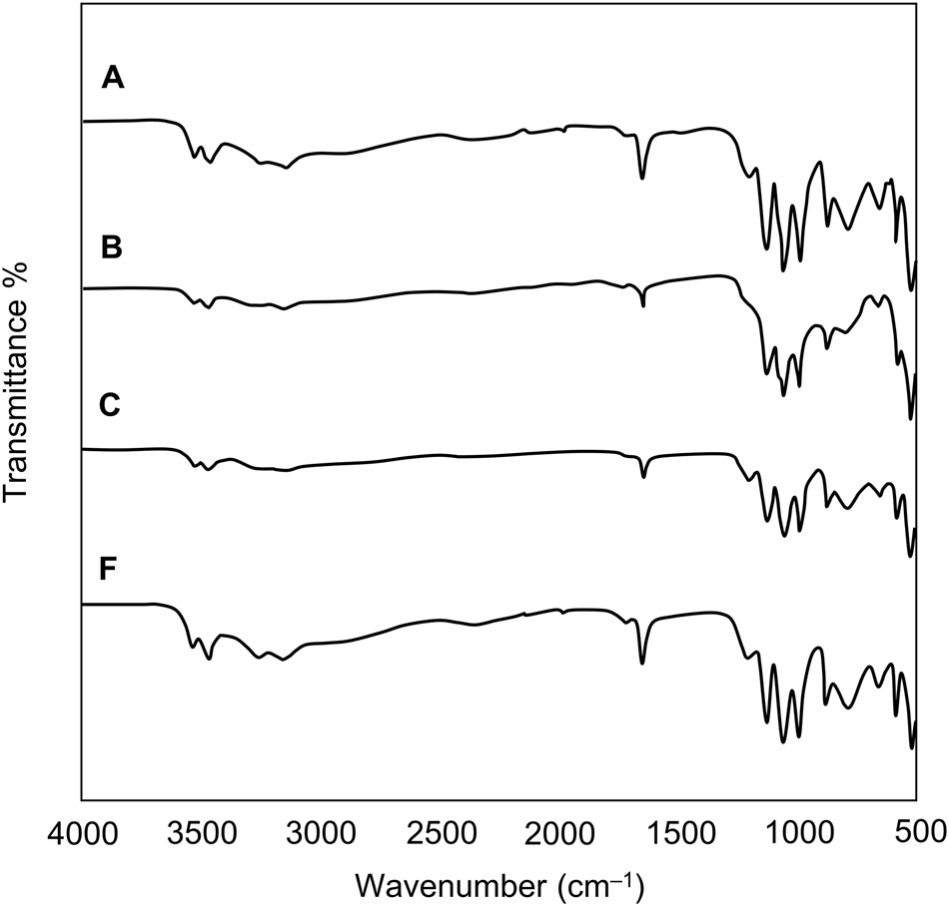
FTIR spectra of samples prepared in experiments (**A**) growth of DCPD on DCPD seed material; **B** growth of DCPD on DCPD seed material in the presence of alanine; **C** growth of DCPD on DCPD seed material in the presence of glutamic acid and F: spontaneously grown DCPD (labile growth zone)

FTIR spectra of DCPD materials synthesised under the various conditions are shown in Fig. 6. All spectra display bands characteristic of DCPD as given above under characterisation of seed materials. A reduction in resolution of the doublet in the 3300–3100 cm^−1^ region suggests that there is a decrease in the crystallinity of materials produced in the presence of both alanine and glutamic acid. Samples A and F showed Raman spectra with spectral characteristics almost identical to that given in Fig. 2, including a major reflection at 985 cm^−1^, indicating that DCPD had formed. Note that ~300 mg product was obtained in addition to seed material for sample A; 200 mg for sample B; 20 mg for sample C and negligible amounts for samples D and E. Raman spectra of samples synthesised in the presence of organic modifiers could not be obtained due to fluorescence of the samples.

### XRD analysis

**Fig. 7:**
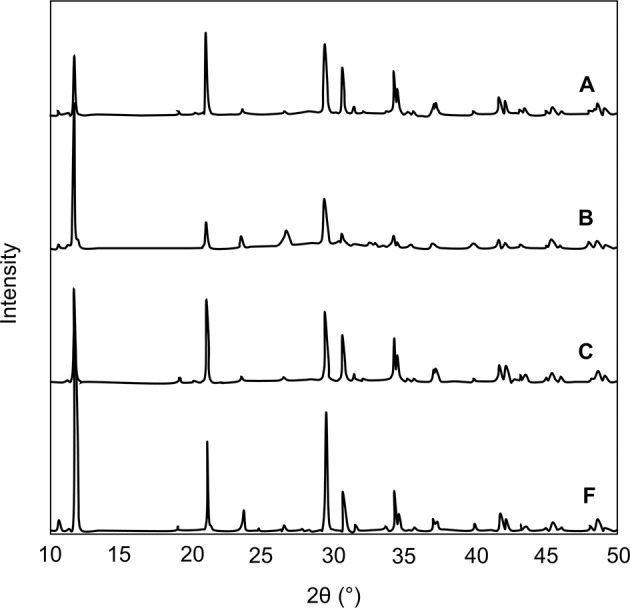
Powder XRD patterns of samples prepared in experiments **A** growth of DCPD on DCPD seed material; **B** growth of DCPD on DCPD seed material in the presence of alanine; **C** growth of DCPD on DCPD seed material in the presence of glutamic acid and F: spontaneously grown DCPD (labile growth zone)

Powder XRD patterns of DCPD materials synthesised under the various conditions are shown in Fig. 7. All diffraction peaks corresponded to DCPD, and no additional CaP phases were identified, indicating that DCPD overgrowth has most likely proceeded via an epitaxial process. In general, differences in peak broadness originating from decreased or increased crystallinity could not be discerned, except for sample B where some broadening of peaks was apparent.

### SEM studies

**Fig. 8:**
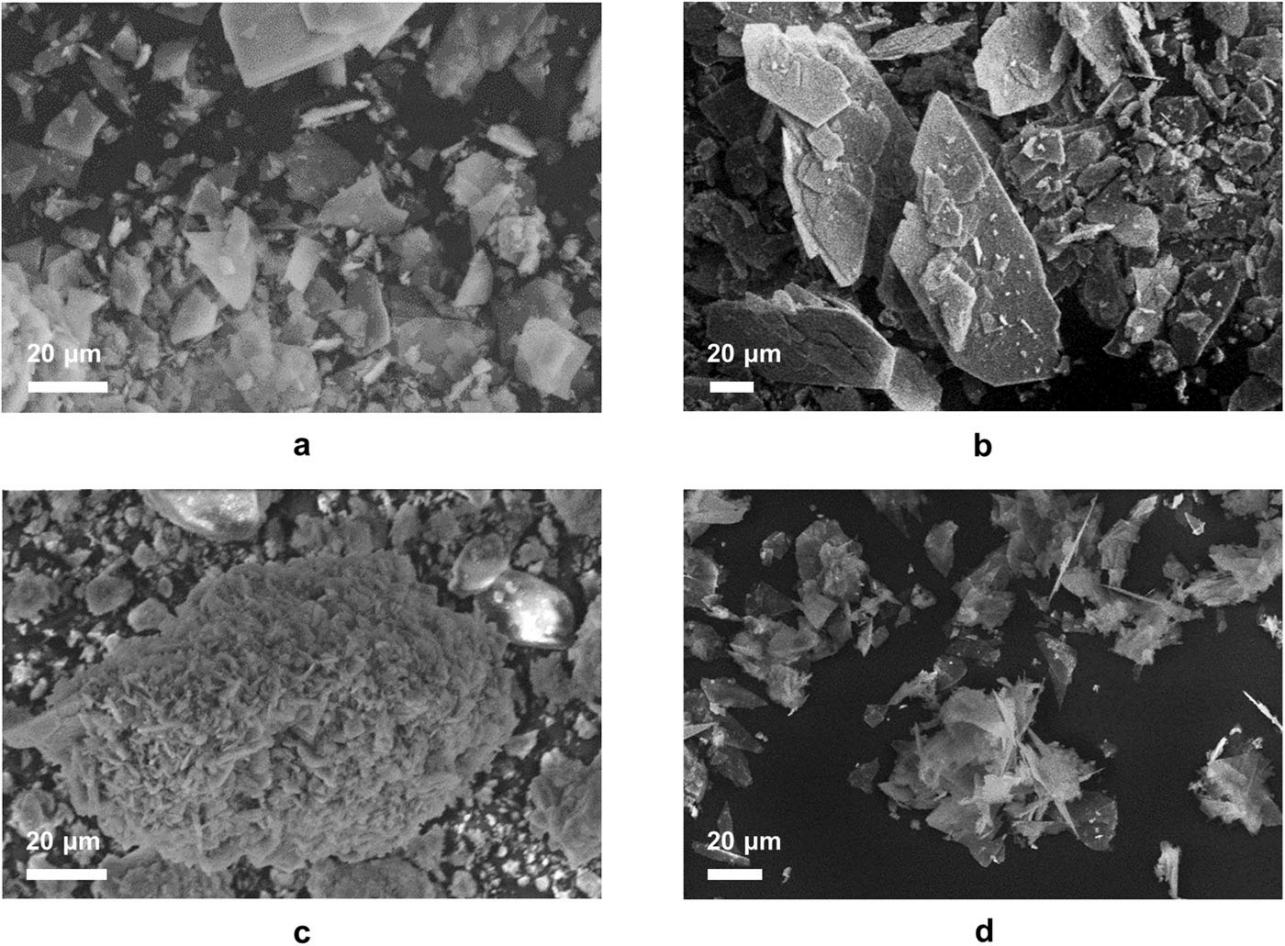
SEM images of (**a**) DCPD grown on DCPD seed material (**b**) DCPD grown on DCPD seed in the presence of alanine (**c**) DCPD grown on DCPD in the presence of glutamic acid and (**d**) DCPD grown under spontaneous conditions (labile zone)

SEM images for materials prepared in experiments A to C (metastable zone), together with experiment F (labile zone) are shown in Fig. 8. The image for DCPD-F displays the characteristic plate-like morphology of crystalline DCPD (compare with Fig. 4). However, samples A and B show a variety of both platey and irregular morphologies. The SEM image for DCPD-C (seed + glutamic acid) displays plate-like crystals that have clustered into chrysanthemum-like aggregates.

## Discussion

This study is one of only a very few to investigate the role of amino acid side-chain polarity on the formation of DCPD from metastable rather than supersaturated solutions and at constant pH [23]. By inhibiting growth, crystals are forced to grow with unusual morphologies such as that seen here in the presence of glutamic acid (Fig. 8c). Crystal growth may then be further inhibited as ions will not be able to interact easily with crystals in clustered arrangements.

The mechanism of stone formation is complex and lacks complete understanding; however formation will proceed generally through a process of crystal nucleation, growth and aggregation. Urine is metastable with respect to calcium oxalate and in some cases CaP [24]. Spontaneous precipitation of COM is unlikely from metastable urine. It is hypothesised that DCPD initially precipitates and subsequently acts as a nidus for COM growth. Randall’s plaques are CaPs deposited in the renal papilla (where urine is emptied into the kidney) and are thought to be sites of urinary stone formation. Plaques are most commonly composed of carbonated apatite, however in rare cases DCPD has been identified as the dominant phase. Studies have suggested that DCPD grows initially, before transforming to the more thermodynamically stable apatite, explaining the detection of apatite [9, 24, 25]. Tang et al. have shown that in vitro DCPD preferentially nucleates from mildly acidic urine, supersaturated with respect to COM, before undergoing dissolution during COM crystal growth [9]. Species that prevent stone formation, including small molecules like citrate and pyrophosphate, may be present [26]. Inhibitory species also include large macromolecules such as glycoproteins, glycosaminoglycans and proteoglycans. Several of these urinary macromolecules contain polar amino acids and phosphorylated residues. For example, urinary prothrombin fragment 1 contains γ-carboxyglutamic acid while osteopontin includes aspartic acid and may contain phosphorylated serine and threonine. In instances of stone formation, it has been suggested that these macromolecules may be deficient or defective [26]. While the inhibitory roles of these proteins have been extensively researched, paradoxical effects whereby macromolecules inhibit and promote crystal growth, indicate the need for continued research.

Amino acids are thought to inhibit the growth of DCPD by an adsorption mechanism. The mechanism is governed by electrostatic interactions between amino acids side groups, and ions present on CaP nuclei and seed material. Complexes can also form between amino acids and free calcium and phosphate ions in solution [11]. In the current study, alanine was found to be the weakest inhibitor of DCPD (Fig. 5B). At pH 5.0, alanine has a net charge of zero, and thus interactions between the molecule and charged calcium and phosphate species are unlikely [27].

The pKa value for the carboxylic acid side group on glutamic acid is ~4.2. Hence, at pH 5.0, dissolved glutamic acid would be present in both zwitterionic and negatively charged conformations. Of these, the negatively charged form would predominate. Studies have suggested that carboxylic acid side groups adsorb onto Ca^2+^ ions found at kinks and dislocations on the surface of DCPD [23]. These authors have also suggested that the zwitterionic form could adsorb onto DCPD, by redistribution of electron density in carboxylic acid side groups to oxygen, thus causing a negative charge. Polar side groups thus contribute to the inhibitory abilities of amino acids and may explain why alanine, which contains a non-polar methyl side group, did not effectively inhibit DCPD growth (Fig. 5).

**Fig. 9:**
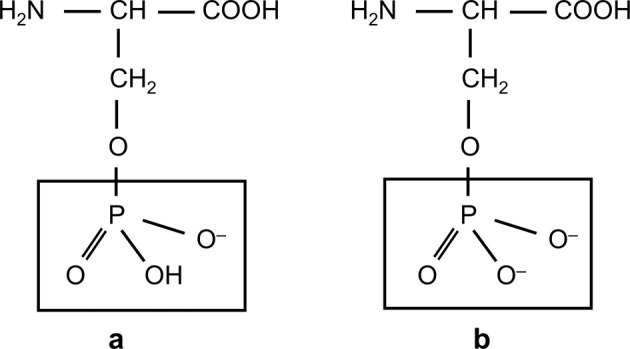
Molecular structures for (**a**) singly and (**b**) doubly charged phosphoserine

In the case of phosphoserine, it is hypothesised that the negatively charged side groups of phosphoserine adsorb onto seed material in solution, inhibiting the ability of solution precipitation onto substrates. In the absence of seed material, nucleation would have to be spontaneous, which is extremely unlikely in metastable solutions. The adsorption affinity of phosphoserine compared with glutamic acid can be explained by their relative charges. Xie et al. have shown that phosphoserine transforms from a singly to doubly charged structure (Fig. 9) in response to changing pH. Raman bands at 980 and 1080 cm^−1^ correspond to singly and doubly charged phosphate groups in phosphoserine. By use of Raman spectroscopy these authors were able to determine which form was present at specific pH values [28]. At pH 5.0 they showed that both charged forms are present. The doubly charged phosphoserine group contains two deprotonated hydroxyl groups, giving an overall charge of 2−, thus causing stronger electrostatic interactions with DCPD than glutamic acid, which carries a 1− or neutral charge.

CPP is thought to inhibit CaP growth through an adsorption mechanism, similar to that of amino acids [29]. An increase in negatively charged phosphate groups, such as is found in polyphosphoserine, should enhance the inhibitory ability of the molecule and be a possible candidate for treatment and prevention of pathogenic CaPs.

## Conclusions

1. An understanding of the role of amino acids on DCPD mineralisation could lead to the design of new therapies to target mineralisation-related disorders such as dental calculi and kidney and urinary stones.

2. The results of this study show that amino acids with polar side chains have the greatest inhibitory effect on the growth of DCPD on seed material from metastable solutions. Doubly charged phosphorylated side chains are more effective inhibitors than singly charged carboxylated side chains.

3. Inhibition of crystal growth most likely proceeds via an adsorption mechanism. When this happens, crystals with unusual morphologies may develop leading to further inability of ions to interact with crystals in these arrangements.
